# Variable In Vivo Hepatitis D Virus (HDV) RNA Editing Rates According to the HDV Genotype

**DOI:** 10.3390/v13081572

**Published:** 2021-08-09

**Authors:** Samira Dziri, Christophe Rodriguez, Athenaïs Gerber, Ségolène Brichler, Chakib Alloui, Dominique Roulot, Paul Dény, Jean Michel Pawlotsky, Emmanuel Gordien, Frédéric Le Gal

**Affiliations:** 1 Centre National de Référence des Hépatites Virales B, C et Delta, Laboratoire de Microbiologie Clinique, Hôpital-Avicenne, Assistance Publique Hôpitaux de Paris, Université Sorbonne Paris Cité, 93000 Bobigny, France; samira.dziri@aphp.fr (S.D.); athenais.gerber@aphp.fr (A.G.); segolene.brichler@aphp.fr (S.B.); chakib.alloui@aphp.fr (C.A.); paul.deny@inserm.fr (P.D.); emmanuel.gordien@aphp.fr (E.G.); 2 Centre National de référence des Hépatites Virales B, C et Delta, Département de Virologie, Hôpital Henri Mondor, Assistance Publique-Hôpitaux de Paris, Université Paris-Est, 94000 Créteil, France; christophe.rodriguez@aphp.fr (C.R.); jean-michel.pawlotsky@aphp.fr (J.M.P.); 3 Unité INSERM U955, équipe 18, 94000 Créteil, France; dominique.roulot@aphp.fr; 4 Unité d’hépatologie, Hôpital Avicenne, Assistance Publique-Hôpitaux de Paris, Université Sorbonne-Paris-Cité, 93000 Bobigny, France; 5 Centre de Recherche en Cancérologie de Lyon, INSERM U1052-UMR CNRS 5286, 69001 Lyon, France

**Keywords:** HDV, editing, genotype, HDAg, next-generation-sequencing, pathogenesis

## Abstract

Human hepatitis delta virus (HDV) is a small defective RNA satellite virus that requires hepatitis B virus (HBV) envelope proteins to form its own virions. The HDV genome possesses a single coding open reading frame (ORF), located on a replicative intermediate, the antigenome, encoding the small (s) and the large (L) isoforms of the delta antigen (s-HDAg and L-HDAg). The latter is produced following an editing process, changing the amber/stop codon on the s-HDAg-ORF into a tryptophan codon, allowing L-HDAg synthesis by the addition of 19 (or 20) C-terminal amino acids. The two delta proteins play different roles in the viral cell cycle: s-HDAg activates genome replication, while L-HDAg blocks replication and favors virion morphogenesis and propagation. L-HDAg has also been involved in HDV pathogenicity. Understanding the kinetics of viral editing rates in vivo is key to unravel the biology of the virus and understand its spread and natural history. We developed and validated a new assay based on next-generation sequencing and aimed at quantifying HDV RNA editing in plasma. We analyzed plasma samples from 219 patients infected with different HDV genotypes and showed that HDV editing capacity strongly depends on the genotype of the strain.

## 1. Introduction

Hepatitis delta virus (HDV) is a small defective RNA virus that infects humans already chronically carrying hepatitis B virus (HBV). Indeed, HDV requires the HBV envelope proteins expressing the HBV surface antigen (HBsAg) for the morphogenesis of its viral particles [1]. HDV has been assigned to the *Deltavirus* genus. New HDV RNA-like sequences have been recently identified in the animal kingdom [2,3,4,5]. Human HDV infection is widespread, and its prevalence differs between areas. It is estimated that, among the 257 million individuals with chronic HBV infection, approximately 15–20 million are also infected with HDV. According to recent meta-analyses, this number may be underestimated in several countries [6,7]. HDV infection is associated with the most severe forms of viral hepatitis, ranging from acute and sometimes fulminant disease to a rapidly progressive form of chronic viral hepatitis rapidly leading to cirrhosis and hepatocellular carcinoma [8].

The HDV genus is characterized by a very high genetic diversity. We recently proposed, based on large-scale sequencing of HDV strains for many regions of the world, individualizing eight HDV genotypes, numbered HDV-1 to HDV-8, that differ by ≤20% over the sequence of their full-length genome, with a strong robustness of branch topologies in Bayesian-based tree reconstruction [9,10]. HDV genotypes can be further split into two or more subgenotypes that differ by ≤10% of their sequence (≤16% for HDV-1) [9]. Interestingly, HDV genotypes and subgenotypes have a distinct worldwide geographic distribution [9,10,11]. 

The HDV virion is approximately 36 nm in diameter. The viral particle is composed of a double lipid layer in which the HBV envelope glycoproteins are anchored surrounding the HDV ribonucleoprotein (RNP). This RNP is composed of the circular single-stranded RNA of negative polarity containing ~1700 nucleotides (nt) closely linked to the two isoforms of the HDV protein, including the small (s) and the large (L) hepatitis delta antigens (s-HDAg and L-HDAg, respectively) [12]. The HDV genome has a high (approximately 60%) GC content. It is characterized by an intramolecular base-pairing of 74%, which confers the genome an unbranched rod-like structure [9,13].

HDV RNA replication is carried out independently of HBV by the host DNA-dependent RNA polymerase-2 (RNA-Pol-II) [14]. It has been suggested that the RNA-Pol-I and/or RNA-Pol-III can drive genomic-to-antigenomic RNA synthesis in the nucleolus, although this remains debated [15]. The double-rolling circle model, similar to that in plant viroid agents, generates a complimentary copy of the genome, the replication intermediate known as the antigenome [16,17], and numerous copies of the genomic sense RNA synthesized from the antigenomic template in the nucleoplasm. In addition, the genome is also transcribed in a messenger RNA (mRNA) of antigenomic polarity (~0.8 kb) coding for the s-HDAg protein of 195 amino acids.

As replication proceeds, a fraction of the full-length antigenome RNA undergoes editing by a specific host cellular enzyme, the adenosine deaminase acting on double stranded RNA (ADAR), essentially its short isoform ADAR-1 [18,19]. As described for HDV-1, additional RNA structures are required to optimize editing. They include: (I) an A-C mismatch at the editing amber/stop codon (UAG) ending the s-HDAg gene at position 1012 on the antigenome; (II) strict base pairing of four nucleotide pairs surrounding this position in both directions; and (III) a specific secondary structure, located approximately 25 nucleotides 3′ downstream, called the dsRNA binding motif (DRBM), which defines the minimal editing substrate for ADAR-1. Indeed, this structure is required for the initiation of editing [20,21], and we recently showed that it can vary according to the HDV genotype [9]. It has also been shown that a 16/17-nt segment located as far as 114 nt upstream of the editing site of HDV-1 was involved in this process [22]. Thus, ADAR-1 mediates editing of the antigenomic RNA by acting on the adenosine within the amber/stop codon (UAG) of the s-HDAg ORF, which is deaminated into an inosine. Through the replication process, the stop codon (UAG) is changed into a tryptophan codon W (UGG), allowing the extension of the s-HDAg ORF during translation to produce the L-HDAg by the addition of 19 (or 20 for HDV-3) amino acid residues at the C-terminus of the s-HDAg protein. The small protein s-HDAg, while having no polymerase activity, is involved in genome replication through the recruitment of the cellular DNA-dependent RNA polymerase 2 by histone mimicry [23]. In contrast, due to its carboxyl-extension that adds a farnesyl transferase signal, the isoprenylated L-HDAg protein favors virion morphogenesis via a nuclear export signal that may contribute to the cytoplasmic membrane localization of the delta RNP. L-HDAg has also been involved in HDV pathogenicity [24,25,26,27,28,29,30].

In earlier studies [31,32] (and unpublished personal data), Sanger sequencing of PCR amplicons from plasma samples often results in double populations at the amber-stop/W codon position that are difficult to interpret. This finding suggests that both replication-competent and defective quasi-species variants circulate in the peripheral blood of infected individuals. Interestingly, for HDV-1 strains, amplicons spanning the L-HDAg ORF display a specific NcoI restriction site. Restriction analyses in an HDV-infected woodchuck model showed a high and rapid decrease in the proportion of editing clones in the context of a transient viremia. Therefore, hypotheses have been raised as to a role for the early expression of L-HDAg in viral clearance and/or for a better presentation of some HDAg epitopes to the immune system [33]. In a first attempt to use next-generation sequencing (NGS) to characterize samples from three patients studied longitudinally, chronic HDV infection appeared to be associated with a higher proportion of non-edited HDV RNA [34]. More recently, Sopena and colleagues studied four patients infected with HDV-1 strains using the Roche NGS-based assay and confirmed the performance of such an approach compared to cloning followed by Sanger sequencing [35].

The aim of our study was to develop a dedicated tool to accurately quantify HDV-RNA editing rates in vivo by means of NGS, a powerful method for the quantitative characterization of genome populations within complex mixtures and assess the influence of the HDV genotypes on HDV RNA editing. For this, well-characterized samples from 219 untreated patients infected with various HDV genotypes from the French National HDV reference laboratory collection were selected and the editing rates (Edited(E)/Non-Edited (NE) RNA + Edited (E) RNA ratio) were measured. The HDV RNA secondary structures of full-length antigenomes from different genotypes were predicted in silico, with special focus on the editing substrate region.

## 2. Materials and Methods

Implementation and validation of a new tool to quantify HDV RNA editing rates.

To implement a new assay to measure HDV RNA editing rates, we used an NGS ‘method using the MiSeq^®^ Illumina sequencer (Illumina, Eindhoven, The Netherlands) to quantify the proportion of edited versus non-edited genome sequences in the plasma of HDV-infected patients. To analyze the large amount of sequence data generated, we took advantage of the patent-protected “in-house” software recently developed for HIV sequence analyses, including the Pyromute, Pyrotrop and Pyrolink modules, which were further developed to create the PyroEdit software.

Initial validation of the software was performed using computer-generated edited and non-edited sequences from reference sequences. Briefly, we created a program that generated several synthetic fastq files with the following characteristics: (I) fastq files containing 10^3^, 5 × 10^5^ and 10^5^ sequences; (II) consensus sequences from all HDV genotypes (HDV-1 to -8); (III) the presence of the W codon on all sequences of each genotype; (IV) sequence variability up to 30%; (V) Phred Quality Score ≥30, and (VI) a lower limit of detection of the W codon of 1%.

Then, we generated clones (see below) expressing edited and non-edited genome sequences that were mixed at different ratios, respectively: 0%/100%, 5%/95%, 10%/90%, 25%/75%, 50%/50%, 75%/25%, 90%/10%, 95%/5%, 100%/0%. Each different mixture was analyzed 8 times in the same experiment.

Finally, we analyzed the plasma samples from 219 HDV-infected patients described below. Repeatability and reproducibility experiments were conducted from 8 samples, including 3 low (10%), 3 median (32%) and 2 high (52%) RNA editing rates, that were tested 10 times using the same MiSeq^®^ flow cell and 3 times on 3 different MiSeq^®^ flow cells (Illumina, Eindhoven, The Netherlands).

### 2.1. Generation of Non-Edited and Edited Clones

We generated non-edited and edited clones from a well-characterized HDV-1 strain (dFr663d unpublished), which exhibited a double population by Sanger sequencing, ATC (for non-edited) and ACC (for edited) at the 1012 position of the genome corresponding to amber (UAG)/W (UGG) codon for the antigenome. 

Briefly, amplicons of the so-called R0 genome region, spanning nt 920 to 1289 and covering the editing region [9], were generated after reverse transcription using the SuperScriptIII Reverse Transcriptase (Invitrogen by ThermoFisher Scientific, Waltham, MA, USA) and amplification with the KAPA High-Fidelity PCR kit (Roche, Indianapolis, IN, USA). They were then cloned into a TOPO10 vector (Thermofisher Scientific, Waltham, MA, USA) and plasmids were expressed in *E. Coli* bacteria. The final non-edited and edited purified clones were collected, further amplified using the KAPA high fidelity PCR kit and sequenced by the Sanger method using the ABI Prism Big Dye Terminator Cycle Sequencing Kit v3.1 (Applied Biosystems by ThermoFisher Scientific, Waltham, MA, USA). DNA plasmid purification was carried out with the GeneJET plasmid miniprep kit (Thermofisher Scientific, Waltham, MA, USA). DNA was normalized using a Qubit Fluorometer (Qubit dsDNA Assay kit, Life Technologies by ThermoFisher Scientific. Waltham, MA, USA), and new PCRs were performed to characterize each non-edited and edited clones by means of Sanger sequencing. Quantification of each clone by NGS was performed through new PCR amplifications using a pair of primers containing the 5′ NGS-adaptor sequences P5 and P7 (Nextera XT Index Kit V2, Illumina, Eindhoven, The Netherlands). PCR products were further purified using the NXp Station (Agencourt AMPure XP, Beckman Coulter, Indianapolis, IN, USA). DNA quantity and quality were assessed using Qubit and BioAnalyser (Agilent by Thermofisher Scientific, Waltham, MA, USA).

### 2.2. Quantification of the In Vivo Editing Rate in Plasma Samples

Clinical samples from 219 untreated patients infected with different HDV genotypes were selected from the collection of the French HDV National Reference Laboratory according to the HDV genotype and a HDV viral load > 1000 UI/mL. HDV RNA levels ranged between 2.7 and 10.2 Log IU/mL, with a median of 6.3 ± 1.62 Log IU/mL, as quantified by the commercial Eurobioplex HDV kit (Eurobio, Les Ulis, France) [36]. HBV DNA levels, quantified by the Cobas HBV 6800 Systems (Roche, Waltham, MA, USA), were available in 139 of the 219 patients and their values ranged between 1 and 6.6 Log IU/mL, with a median of 1.83 ± 1.94 Log IU/mL, 5% of them being close to the limit of detection of the assay. HDV genotypes, determined as previously described [9,11], were distributed as follows: 115 HDV-1, 55 HDV-5, 12 HDV-6, 17 HDV-7 and 20 HDV-8. 

For the quantification of HDV RNA editing rate, RNAs were extracted from plasma samples with the m2000sp system (Abbott Molecular, Chicago, IL, USA) and reverse-transcribed with the SuperScript III First-Strand Synthesis System (Invitrogen by ThermoFisher Scientific, Waltham, MA, USA) according to the manufacturer’s instructions. The cDNAs were amplified as previously described [9,37] using the KAPA High-Fidelity DNA polymerase (KAPABiosystems, Roche, Indianapolis, IN, USA) and improving proofreading efficiency by using specific primers corresponding to the R0 genome region (See Appendix A, Table A1). After purification and quantification of the PCR products, a library was created containing all samples at the same normalized concentration, which was sequenced by NGS on the MiSeq^®^ System (MySeq^®^ V2 Reagent Kit 500 cycles, Illumina, Eindhoven, The Netherlands) according to the manufacturer’s protocol.

NGS data analysis was performed with our new dedicated tool PyroEdit. After a specific quality filter step, only complete sequences (up to 151 pb) with a Phred Quality Scores >30 were recorded and aligned (Smith–Waterman algorithm) with a genotype-specific consensus reference sequence. The percentage of sequences bearing the W at the amber/W codon was calculated for each patient sample and recorded in the final report. 

### 2.3. Determination of the Secondary Structures of the RNA Editing Region

To analyze secondary HDV RNA structures, especially in the region surrounding the amber/W editing site, we determined the full-length genome sequence of 32 out of the 219 strains from the initial cohort (10 HDV-1, 7 HDV-5, 5 HDV-6, 5 HDV-7 and 5 HDV-8), as previously described [9] (Being published in EMBL). The more stable predicted secondary structures (RNAfolding) of the RNA editing site regions were determined using two online software applications with default setting from the entire antigenome sequences: RNAfold WebServer (http://rna.tbi.univie.ac.at/cgi-bin/RNAWebSuite/RNAfold.cgi accessed on 1 November 2019) and UNAfold WebServer (http://www.unafold.org/mfold/applications/rna-folding-form.php accessed on 1 June 2021). We then compared the patterns of the putative minimum editing substrate for the ADAR-1 enzyme according to the genotype and the editing rate.

### 2.4. Statistical Analyses

Statistical analyses were carried out using the R software (version 3.5.1, 2018 Free Software Foundation, Boston, MA, USA). The non-parametric Kruskal–Wallis test was used to compare population distributions. The Wilcoxon test was used to analyze paired samples of non-categorical data, while Pearson’s test was used for statistical correlations. *p* values of <0.05 denoted statistical significance. Figures were carried out under ggplot2.

## 3. Results

### 3.1. Provision of a New Tool to Quantify HDV RNA Editing Rate

We developed a new tool to measure the editing capacity of HDV strains in samples from infected patients. The workflow is based on NGS and quantifies editing of the amber/stop codon at positions 1010–1013 of the antigenomic RNA.

**Figure 1:**
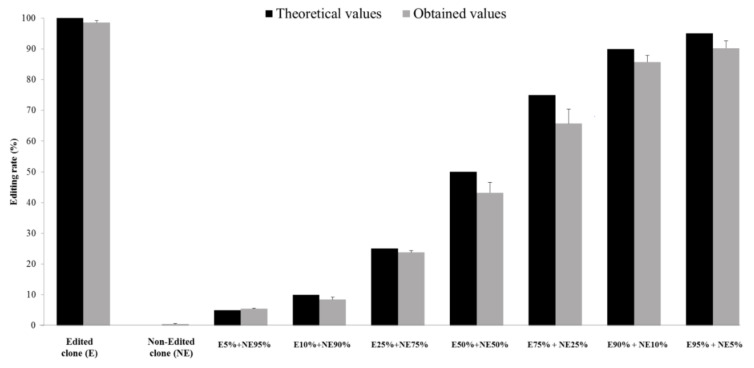
Validation of the editing rate quantification assay. Edited and non-edited clones were generated from an HDV-1 strain. The two clones were mixed in different proportions (see ‘Methods’ section) and analyzed. The expected values (in black) were compared to the observed values (in gray). The standard deviation was calculated from 8 values obtained for each mixture.

Previously created in-house software was adapted to develop the PyroEdit application, that was subsequently validated to be used regardless of the number of sequences to analyze, threshold values, virus variability and HDV genotype. Firstly, the validation process was conducted by using computer-generated fastq files, as described in the “Methods” section. The mean difference between the expected values of edited genome rates (input) and the values calculated by PyroEdit (output) was less than 0.1%, whatever the HDV genotype is (data not shown). Secondly, eight mixtures containing clones expressing either edited or non-edited genomes in various proportions were prepared (Figure 1). A total of 18,400,000 quality-filtered sequences were obtained (mean: 175,792 sequences per sample) with a Phred Quality Score ≥ 30. The results obtained with PyroEdit were very close to the expected values, with a mean difference < 3%. The lower limit of quantification (LLOQ) was 5% (percentage of edited RNA in a mixture) and the lower limit of detection (LLOD), defined by the threshold for detection according to information from Illumina support, was 1% (Figure 1). Furthermore, repeatability and reproducibility were both high (standard deviations: <18.2 and <8.75%, respectively), independent of the editing rate (low, 10%; medium, 30%; high >50%).

### 3.2. In Vivo Editing Rates in Clinical Samples

**Figure 2:**
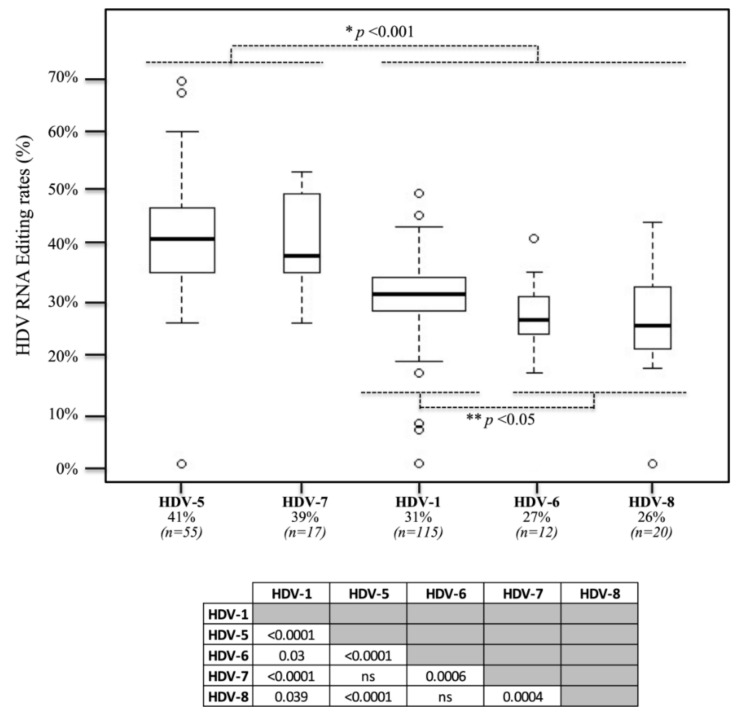
Plasma HDV RNA editing rates according to the HDV genotype. Two-hundred and nineteen samples from untreated infected patients were analyzed. The box plots, generated with ggplot2 application, show the median editing rate and the value distribution obtained with different genotypes. Significantly higher editing rates (expressed in percentage) were observed for HDV-5 and HDV-7 strains, compared to HDV-1 (African and non-African genotypes), HDV-6 and HDV-8 (* *p* < 0.001). In addition, HDV-6 and HDV-8 editing rates were significantly lower than HDV-1 ones (** *p* < 0.05). Comparison of editing rates was performed by means of Kruskal–Wallis, chi-square, and Wilcoxon’s tests.

Editing rates were quantified in plasma samples from 219 patients infected with different HDV genotypes, including HDV-1 (*n* = 115), HDV-5 (*n* = 55), HDV-6 (*n* = 12), HDV-7 (*n* = 17) and HDV-8 (*n* = 20). The editing rates ranged from <5% to 69% (median: 32%). In three samples, the editing rate was repeatedly comprised between the LLOD (1%) and the LLOQ (5%). Higher and lower values were controlled twice, and an identical result was found. The median editing rate values were different across the different HDV genotypes: 31%, 41%, 27%, 39% and 26% for HDV-1, HDV-5, HDV-6, HDV-7 and HDV-8, respectively. The difference was significant between HDV-5 and HDV-7 compared to that between HDV-1, -6 and -8, as assessed by multivariate analyses (*p* < 0.001; Table 1 and Figure 2). In addition, within the latter group, the HDV-1 editing rate was significantly higher than those of HDV-6 and HDV-8 (*p* < 0.05). No significant difference was observed between non-African and African-HDV-1 strains (*n* = 73 and *n* = 42, respectively). Although there was no correlation between the initial HDV viral loads and the editing rate values (Table 1), we found a significant correlation between high editing rate levels and low HBV DNA viral loads (*p* < 0.01).

### 3.3. Predicted RNA Secondary Structures of the Editing Genome Region

**Figure 3:**
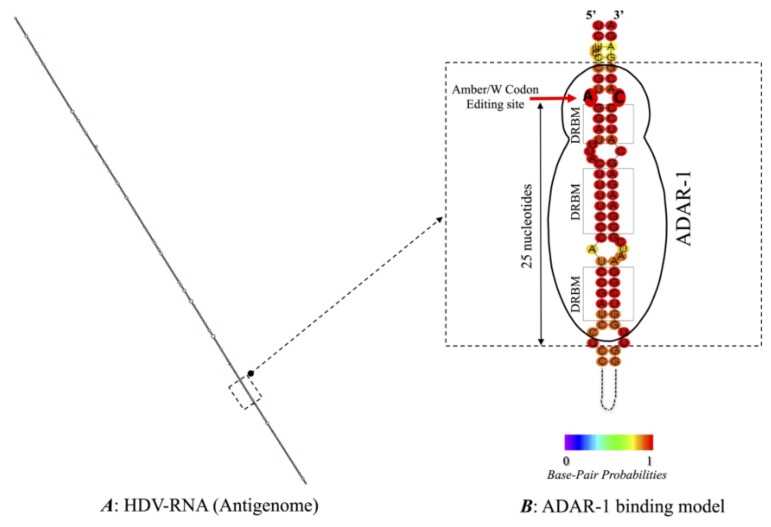
Schematic representation of ADAR-1 binding on the putative minimum editing substrate from the Italian HDV genotype 1 strain (accession number: X0445Italy1976). (**A**) The secondary structure of the complete sequence of antigenomic RNA is shown. The dashed black square delimitates the minimal editing substrate region for host enzyme ADAR-1. (**B**) ADAR-1 binding model on the putative minimum editing substrate. The unbranched rod-like structure with base-pairing domains of the HDV RNA minimal editing substrate is shown. ADAR-1 binding mediated by the 3 dsRNA-binding motifs (DRMBs) is drawn. The red arrow indicates the amber/W editing site with the target adenosine for editing, while the A-C mismatch is highlighted. The color scale indicates relative base-pairing probabilities from 0 (violet) to 1 (red).

**Figure 4:**
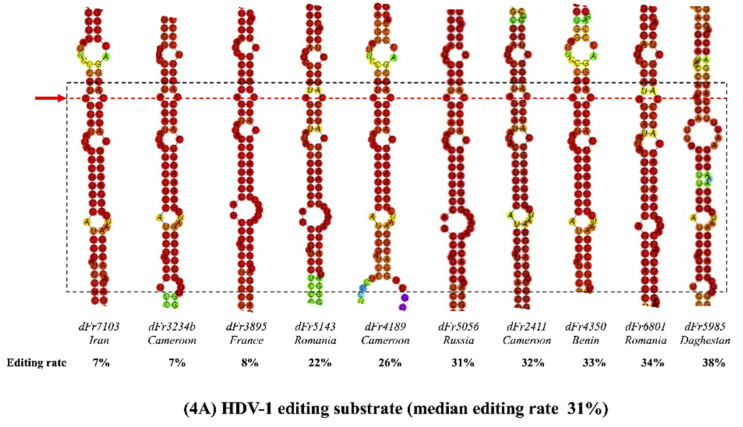

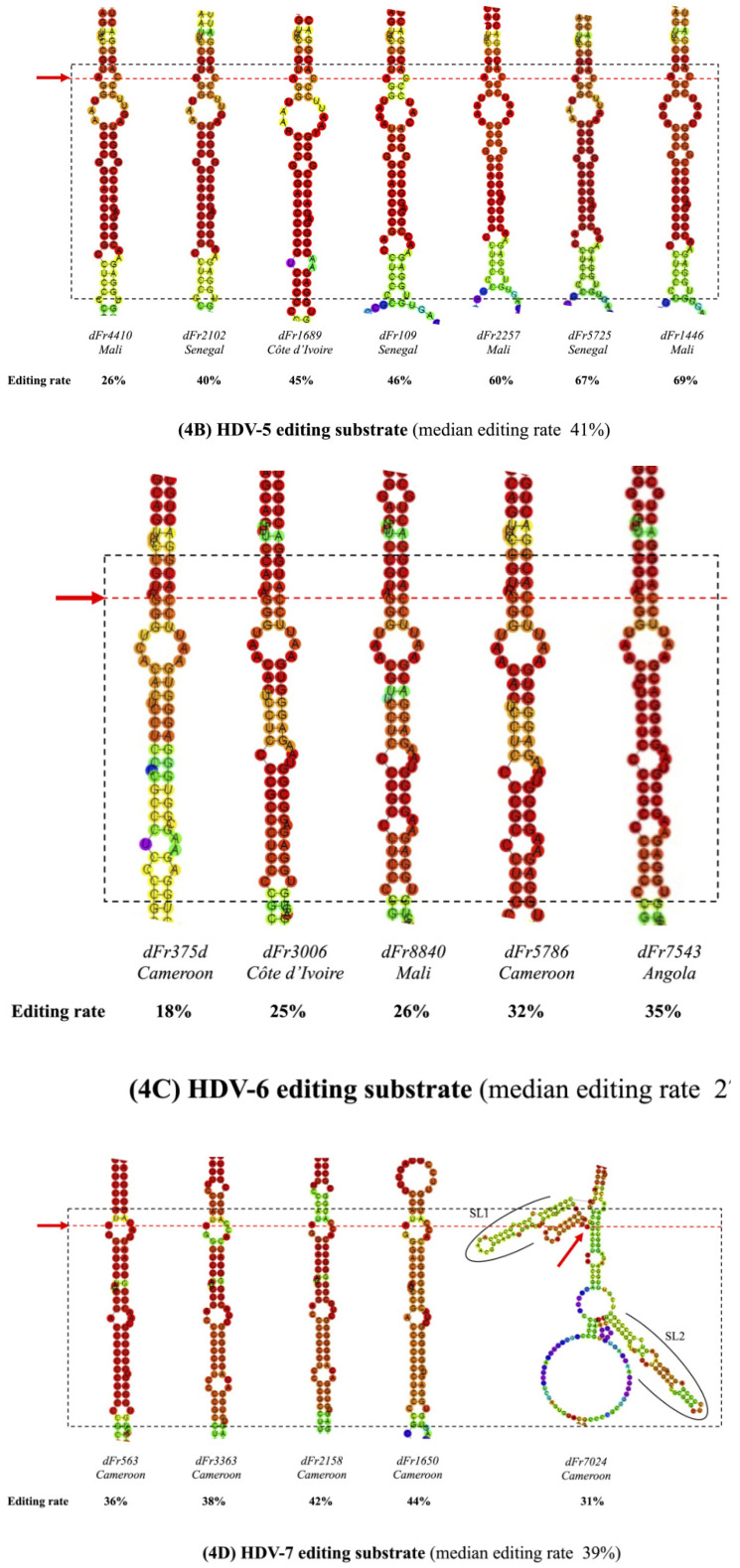

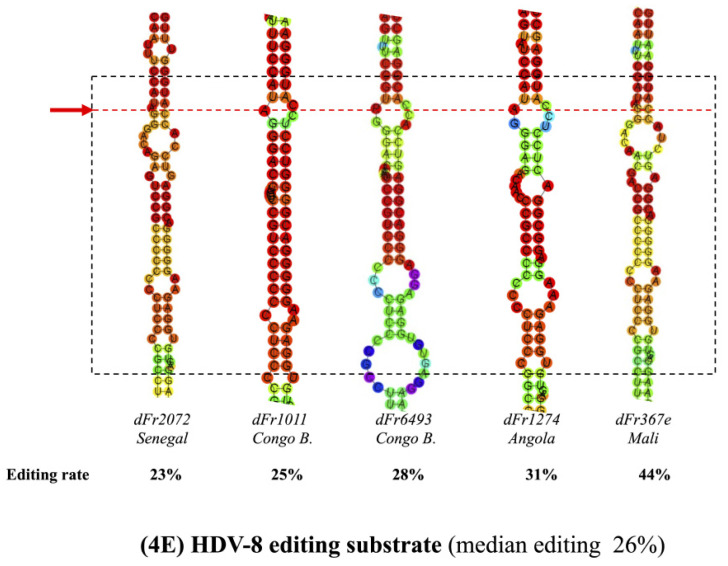
Secondary structures of editing substrate regions from different HDV genotypes: panel (**A**–**E**) for respectively for HDV-1 (10 strains); HDV-5 (7 strains); HDV-6 (5 strains); HDV-7 (5 strains) and HDV-8 (5 strains)**.** These more stable profiles result from the analysis of 32 HDV RNA secondary structures were predicted from full-length antigenome sequences and focus on the amber/W editing site and the 25 downstream nucleotides. The dashed black square delimitates the minimal editing substrate region. The red dashed line corresponds to the amber/W codon and the A-C mismatch at position 1012 of the antigenome. For each strain, its name, country of origin, genome size, free energy to destabilize the structure and editing rate are indicated. The dFr7024 strain (HDV-7; (panel **D**) displays a particular secondary structure in which the unbranched rod-like is rearranged to form to two stem-loops (SL1 and SL2) that flank a central base-pairing region that includes the amber/W site. The color scale indicates the relative base-pairing probabilities from 0 (violet) to 1 (red).

The editing process involves several structural requirements that are described in the introduction. RNA secondary structures can differ according to the HDV genotype, as described earlier [9,21,38]. Indeed, loops of variable sizes are directly displayed at the vicinity of the editing site and downstream, disrupting base pairing that is essential for ADAR-1 binding. We characterized full-length viral genome sequences from 32 of the 219 samples, selected to represent different HDV genotypes and according to different editing rate levels. Then, we analyzed the more stable RNA secondary structures of their antigenomic sequence, with the main focus on the so-called “minimal substrate for editing” (Table 2 and Figure 3). The structures were identical whatever the application used, RNAfold or UNAfold. The 32 different structures according to genotypes are presented based on RNAfold analysis in Figure 4.

Nine out of the 10 HDV-1 sequences analyzed showed similar secondary structure patterns, composed of three long base-pairing motifs and two bulges (Figure 4A). By contrast, analysis of the seven HDV-5 strain sequences showed one short base-pairing motif followed by three long base-pairing motifs, separated by one large bubble and two small bulges (Figure 4B). The five HDV-6 strains displayed three or four base-pairing motifs interspersed with two or three bulges (Figure 4C). As shown in Figure 4D, four out of the five HDV-7 sequences showed a similar pattern, composed of a repetition of two very large base-pairing motifs separated by one bulge. However, the remaining one (dFr7024) displayed a branched structure composed of two stem-loops flanking the base-pairing motif, as has been previously described for HDV-3 [19,39], with a same free energy than the other HDV-7 strains. Finally, the five HDV-8 strains showed no particular structural characteristics (Figure 4E). The presence of an A-C mismatch at the amber/stop codon editing site has been reported to be related to the editing efficiency of genotype HDV-1. This mismatch was found in HDV-1 and HDV-5 strains in our work and was replaced in HDV-7 strains by a large bubble at the same location. This A-C mismatch pair was absent in HDV-6 and in most HDV-8 strains, suggesting an alternative transient conformation for the editing site. The base located immediately 3′ downstream of the adenosine, known to influence editing efficiency, was always a guanine, whatever the HDV genotype. Of note, however, the number of dsRNA-binding motifs (DRBMs) did not seem to be associated with the editing capacity (Table 2). Finally, when considering the 130 nucleotides located upstream of the amber/W codon in these 32 full-length sequences, we did not find any genotype-specific pattern [22].

## 4. Discussion

RNA editing is responsible for the switch from viral RNA replication to virion packaging and secretion. Indeed, early during the replication cycle, the virus produces the s-HDAg protein, which is required for RNA synthesis as it ensures the recruitment of BAZ2B-associated chromatin remodeling factors (BRFs) on the viral RNA by histone mimicry, favors DNA-dependent RNA Pol 2 hijacking, and allows the Pol2 RNA elongation process to occur [23,40]. At later times in the life cycle, the virus produces the L-HDAg as a result of editing at the amber/W codon, which inhibits RNA synthesis and favors virion morphogenesis. HDV genome replication is highly dependent on the respective proportions of the two forms of HDAg. Indeed, it has been shown in vitro that replication was reduced by as much as 8-fold at a 10:1 s-HDAg/L-HDAg ratio [41,42]. Altogether, HDV RNA editing is a key step in the virus life cycle that plays an important role in the natural history and clinical outcomes of human infection.

In this study, we report the development and validation of a new NGS-based assay using the MiSeq^®^ Illumina device, together with a new in-house software, PyroEdit, which allowed us to measure the viral editing rate in plasma samples from treatment-naïve patients infected with different HDV genotypes. We also analyzed the predicted RNA structural features of the editing region of several strains from this cohort according to editing rate and HDV genotype.

Our results show that our assay was able to: (I) be robust in spite of the high variability of the HDV genome, characterized by a nucleotide dissimilarity rate ranging from 20% to up to 35% over the entire genome sequence between the different HDV genotypes; (II) individualize the sequence of interest of 251 nt in length with a Phred Quality Score > 20% and with 99% accuracy; (III) correctly align each consensus sequence; (IV) check which nucleotide was present at the amber/W codon position and characterize the codon; and (V) calculate the editing rate and provide a final report. With this quantitative method, the editing rate values in clinical samples ranged from 7% to 69% in patients infected with HDV genotypes HDV-1, -5, -6, -7 and -8. Previous in vitro (cell culture) and in vivo (plasma) studies using HDV-1 strains found editing rates around 40% [34,35,43]. Remarkably, in three samples from our study, we repeatedly observed very low editing rate values, lying between the LLOD (1%) and the LLOQ (5%). These results indicate that very low amounts of L-HDAg are sufficient for virion production. At the opposite, strains with high HDV-RNA editing capacity (for instance HDV-5 strains which have a 69% editing capacity), the presence of 31% of non-edited RNA encoding s-HDAg appears to be sufficient for the initiation and continuation of genome replication and to ensure the subsequent steps of the viral life cycle.

Another important result of our study is that the editing rate values differed significantly between different HDV genotypes. Indeed, the African HDV-5 and HDV-7 strains showed significant higher in vivo rates of editing than HDV-1, HDV-6 and HDV-8 (40% vs. 28%, *p* < 0.001). In addition, HDV-1 strains exhibited significantly higher editing capacities than HDV-6 and HDV-8 (*p* < 0.05). Interestingly, in the HDV-1 group, no difference in editing efficiency was seen between African and non-African strains. Because HDV-1 is the most frequent, ubiquitously distributed genotype worldwide, this suggests that the in vivo HDV-1 editing rate, around 30%, could be optimal for a most efficient spreading fitness, with an optimal early-to-late phase of HDAg production ratio.

Based on our predicted structural analyses, our results are supported by at least two RNA structural features described in Figure 4 and Table 2. First, the A-C mismatch pair at the amber/W site, which is key in the editing process of HDV-1 strains, is also present in HDV-5 and HDV-7 strains. Interestingly, this mismatch is accentuated in the HDV-7 amber/W codon, because it is located at the top of a large bulge, whereas the mismatch is disrupted in HDV-6 and some HDV-8 sequences which have the lowest editing rates. In addition, in the 25 nt-long-region downstream the editing codon, we found two or three strict base-pairing motifs, allowing for optimal binding of the host ADAR-1 editing enzyme. Surprisingly, the HDV-7 editing motif of one strain (dFr7024 strain) displayed a complex branched structure composed of two stem-loops flanking a base-pairing motif. The editing capacity of this strain appeared to be lower than that of the four other HDV-7 strains (Figure 4D). Such a structure with two stem-loop arms, has been previously proposed to be a transient secondary structure of the HDV RNA used by HDV-3 strains to improve their editing efficiency by ADAR-1 [19,20,21,39]. Therefore, we hypothesize that this conformation observed in a single HDV-7 strain could be optimal for its editing capacity. Whether different genotypes can transiently use this alternative conformation to up- or down-regulate their RNA editing efficiency remains to be determined. Interestingly, HDV-6 and HDV-8 strains, which did not display the A-C mismatch pair at their amber/W site, could use such transient conformation to increase the binding efficiency of host ADAR-1 to its substrate.

L-HDAg synthesis and its accumulation in several cell compartments has been shown to interfere with multiple cellular signaling pathways and alter the pathogenesis of the infection [24,25,26,27,28,29,30]. Indeed, the overexpression of L-HDAg induces the transactivation of a variety of heterologous promoters and upstream regulatory elements, notably by activating serum response factor-associated transcription [24,25]. Park et al. showed that L-HDAg is able to increase TNF-alpha-induced NF-kappa B transcriptional activation involved in inflammation pathways [27]. In a previous work, we showed that L-HDAg induces NADPH oxidase-4 gene expression, resulting in an increase in cellular reactive oxygen species production, subsequently leading to oxidative stress and activation of pleiotropic transcription factors, including STAT-3 or NF-kappa B [28]. Choi et al. showed that L-HDAg modulates transforming growth factor-beta signaling cascades, arguing for a possible induction of liver fibrosis [26]. Interestingly, we recently confirmed in a large clinical study that patients infected with HDV-5 strains (*n* = 147) were more at risk of developing cirrhosis than those infected with other African genotypes, such as HDV-1 [8]. We hypothesize that the high HDV-5 editing rates observed in vivo in this study could contribute to this observation, encouraging additional experimentations comparing HDV-1- and HDV-5-infected patients to confirm this hypothesis.

It has been reported that HDV proteins (s- and L-HDAg) are involved in immune-mediated liver damage [44]. Data from in vitro experiments and in the chimpanzee model suggested a direct cytopathic effect of s-HDAg on hepatocytes, while L-HDAg epitopes render hepatocytes more susceptible to immune-mediated damages. Thus, cells expressing high levels of L-HDAg are likely to be preferentially lysed by the immune system [45,46]. As a result, monitoring the relative production of HDV proteins by quantifying the RNA editing rate could be relevant for patient management to monitor the natural history of the disease associated with chronic infection.

It has been demonstrated in cell culture experiments that L-HDAg is involved in the inhibition of HBV replication observed in HBV-HDV co- or super-infected individuals [47]. Activation of the human interferon alpha inducible MxA protein was one of the possible explanations for this phenomenon [28,29]. Interestingly, we found a significant relationship between high editing rates leading to L-HDAg synthesis in large amounts and low HBV DNA viral loads in vivo.

Another known consequence of HDV RNA editing is the existence of HDV particles containing edited genomes. Such particles are unable to be at the origin of full viral replication cycles. In the event of a co-infection with virions containing genomes capable of coding for both proteins, L-HDAg would likely inhibit the replication of otherwise competent genomes. With our assay, we found that up to 69% of viral particles contained such edited genomes. These edited particles could behave as defective interfering particles (DIPs), as described for HBV and several other viruses [48]. DIPs are thought to serve as decoys for the immune system, being targeted by immune effectors and/or lowering the expression of their own genes. As a result, they can persist and favor fibrogenesis, inflammation and carcinogenesis. Such a mechanism remains to be studied in chronic HDV infection.

The s-HDAg/L-HDAg ratio and its regulation govern the switch from genome replication to packaging, which seems to be unrelated to the HDV viral levels, as we found no clear relationship between viral loads and editing rates. New treatments against HDV will be available soon in addition to pegylated interferon alpha, including Myrcludex, Lonafarnib, Replicor and others in clinical development. Longitudinal studies assessing the kinetics of HDV editing rates on and after treatment could be particularly interesting to better understand their mechanisms of success and failure. The NGS-based method we have developed appears to be particularly well-suited to this endeavor. In addition, the assay will be very useful to finely characterize the editing process control during the natural history of the infection, both in in vitro and in in vivo studies.

In conclusion, our study provides a new specific tool dedicated to the in vitro or in vivo quantification of HDV editing rates and novel insights into the genotype dependency of this important step of the HDV life cycle. This tool will be useful in large-scale clinical studies in the future to assess the role of editing rates as a predictive marker of the natural history and treatment response of the infection.

## Figures and Tables

**Table 1:** Pearson test correlation analysis performed with R (version 3.5.1, 2018).

Parameters	*p* Value
% Editing/HDV genotype	0.0038
% Editing/HBV viral load	0.01
% Editing/HDV viral load	ns

*p* value: Wilcoxon test. ns: non-significant (>0.05).

**Table 2:** Structural features of the minimal editing region inside the 25 nucleotides 3′-downstream of the editing site according to the HDV genotype.

Strain	HDV Genotype	Editing (%)	A-C Mismatch *	DRMB †	Length #
dFr7103	1	7	Yes	3	4-8-12
dFr3234b	1	7	Yes	4	4-8-7-4
dFr3895	1	8	Yes	3	3-8-21
dFr5143	1	22	Yes	3	4-8-12
dFr4189	1	26	Yes	4	4-8-7-9
dFr5056	1	31	Yes	3	4-8-21
dFr2411	1	32	Yes	3	4-8-11
dFr4350	1	33	Yes	3	4-8-21
dFr6801	1	34	Yes	3	4-8-22
dFr5985	1	38	No	4	3-8-7-4
dFr4410	5	26	Yes	4	2-4-9-4
dFr2102	5	40	Yes	5	2-4-9-4-2
dFr1689	5	45	Yes	4	2-3-9-4
dFr109	5	46	Yes	4	2-3-8-4
dFr2257	5	60	Yes	4	2-3-9-4
dFr5725	5	67	Yes	5	2-4-8-4-1
dFr1446e	5	69	Yes	5	2-3-9-4-1
dFr375d	6	18	No	4	2-13-1-4
dFr3006	6	25	No	4	2-7-9-15
dFr8840	6	26	No	5	2-7-4-4-15
dFr5786	6	32	No	5	2-7-4-4-16
dFr7543	6	35	No	5	2-7-4-4-15
dFr563	7	36	Yes	3	9-10-16
dFr3363	7	38	Yes	3	9-7-8
dFr2158	7	42	Yes	3	9-7-8
dFr1650	7	44	Yes	4	9-10-2-4
dFr2072	8	23	No	5	2-2-9-4-11
dFr1011e	8	25	Yes	3	15-4-9
dFr6493	8	28	Yes	3	12-4-5
dFr1274	8	31	Yes	4	4-7-4-8
dFr367e	8	44	No	5	2-2-8-4-15

(*) Presence or not of the A-C mismatch at the 1012 amber/W position. (†) Number of DRBMs inside the 25 nucleotides 3′-downstream of the editing site. (#) Number of consecutive nucleotides base-pairing for each DRBM.

## Data Availability

Not applicable.

## Appendix A

**Table A1:** Primers used in the different RT-PCR experiments.

Reaction	Primer	Sequence (5′-3′)
RT	Delta 920 fw	CATGCCGACCCGAAGAGGAAAG
	Delta 1286 rv	GAAGGAAGGCCCTCGAGAACAAGA
PCR	Delta 920 fw	TCGTCGGCAGCGTCAGATGTGTATAAGAGAAG
		GCATGCCGACCCGAAGAGGAAAG
	Delta N920 fw	TCGTCGGCAGCGTCAGATGTGTATAAGAGAAG
		NCATGCCGACCCGAAGAGGAAAG
	Delta NN920 fw	TCGTCGGCAGCGTCAGATGTGTATAAGAGACA
		GNNCATGCCGACCCGAAGAGGAAAG
	Delta NNN920 fw	TCGTCGGCAGCGTCAGATGTGTATAAGAGACA
		GNNNCATGCCGACCCGAAGAGGAAAG
	Delta N1289 rv	GTCTCGTGGGCTCGGAGATGTGTATAAGAGAC
		AGNGAAGGAAGGCCCTCGAGAACAAGA

Primers were defined from previous studies [9], allowing to amplify the *R0* region of HDV genome (nt 920 to 1289) encompassing the editing region. Fw (forward); rv (reverse).
